# IL-6 and TNF-α responses to acute and regular exercise in adult individuals with multiple sclerosis (MS): a systematic review and meta-analysis

**DOI:** 10.1186/s40001-022-00814-9

**Published:** 2022-09-26

**Authors:** Parnian Shobeiri, Homa Seyedmirzaei, Nastaran Karimi, Fatemeh Rashidi, Antônio L. Teixeira, Serge Brand, Dena Sadeghi-Bahmani, Nima Rezaei

**Affiliations:** 1 School of Medicine, Children’s Medical Center Hospital, Tehran University of Medical Sciences (TUMS), Dr. Qarib St., Keshavarz Blvd, Tehran, 14194 Iran; 2 Network of Immunity in Infection, Malignancy and Autoimmunity (NIIMA), Universal Scientific Education and Research Network (USERN), Tehran, Iran; 3 Research Center for Immunodeficiencies, Pediatrics Center of Excellence, Children’s Medical Center, Tehran University of Medical Sciences, Tehran, Iran; 4 Non–Communicable Diseases Research Center, Endocrinology and Metabolism Population Sciences Institute, Tehran University of Medical Sciences, Tehran, Iran; 5 Interdisciplinary Neuroscience Research Program (INRP), Tehran University of Medical Sciences, Tehran, Iran; 6 School of Medicine, Sari Branch, Islamic Azad University, Sari, Iran; 7 Department of Psychiatry and Behavioral Sciences, McGovern Medical School, Neuropsychiatry Program, The University of Texas Health Science Center at Houston, Houston, TX USA; 8 Psychiatric Clinics, Center for Affective, Stress and Sleep Disorders, University of Basel, Basel, Switzerland; 9 Division of Sport Science and Psychosocial Health, Department of Sport, Exercise and Health, Faculty of Medicine, University of Basel, Basel, Switzerland; 10 Sleep Disorders Research Center, Kermanshah University of Medical Sciences, Kermanshah, Iran; 11 Substance Abuse Prevention Research Center, Kermanshah University of Medical Sciences, Kermanshah, Iran; 12 Department of Psychology, Stanford University, Stanford, CA USA; 13 Department of Immunology, School of Medicine, Tehran University of Medical Sciences, Tehran, Iran

**Keywords:** Multiple sclerosis, Cytokine, Interleukin-6, TNF-α, Exercise

## Abstract

**Background:**

In both the general population and people with multiple sclerosis (PwMS), physical exercise is associated with improved mental well-being. Moreover, there is evidence of the possible protection of physical activity against disease progression in multiple sclerosis (MS). However, the question arises if acute or regular exercise has any impact on the immune system in PwMS. To answer this question, we performed a systematic review and meta-analysis on both plasma and serum cytokine levels (IL-6 and TNF-α) before and after acute and regular exercise among PwMS and compared to healthy controls.

**Method:**

We performed an online search via PubMed, EMBASE, SCOPUS, Web of Science, and Cochrane Library till September 2021 to identify original studies on IL-6 and TNF-α changes after acute and regular exercise in PwMS and controls. Following the Preferred Reporting Items for Systematic Reviews and Meta-Analyses (PRISMA), 11 original studies were included in the meta-analysis. Sensitivity analyses were used to identify the origins of heterogeneity. R 4.0.4 was used to perform the meta-analysis of IL-6 and TNF-α levels before and after acute and regular exercise in PwMS, compared to controls. This study does not qualify for a clinical trial number.

**Results:**

IL-6 levels did neither increase nor decrease after acute and regular exercise in PwMS, and compared to controls (pre- vs. post-intervention: Standardized Mean Difference (SMD) -0.09, 95% CI [−0.29; 0.11], *p-value* = 0.37, PwMS vs. Control: SMD −0.08, 95% CI [−0.33; 0.16], *p-value* = 0.47). In PwMS, TNF-α levels decreased after regular exercise and when TNF-α levels of both acute and regular exercise were pooled (pre- vs. post-intervention: SMD −0.51, 95% CI [-0.91; 0.11], *p-value* = 0.01, PwMS vs. Control: SMD −0.23, 95% CI [−0.66; 0.18], *p-value* = 0.26). TNF-α levels did neither increase nor decrease after acute and regular exercise in PwMS, when compared to controls.

**Conclusion:**

This systematic review and meta-analysis show that exercise does not lead to significant changes in peripheral levels of IL-6 in PwMS in contrast to the observed response in healthy subjects and other medical contexts. However, regular exercise had a specific anti-inflammatory effect on blood TNF-α levels in PwMS. It remains to be investigated why PwMS display this different exercise-induced pattern of cytokines.

**Supplementary Information:**

The online version contains supplementary material available at 10.1186/s40001-022-00814-9.

## Introduction

Multiple sclerosis (MS) is a chronic autoimmune disease estimated to affect 900,000 people in the United States, and 5–300 per 100,000 people worldwide [1]. It is characterized by varying patterns of neuro-inflammation, demyelination, and axonal loss [2]. As a result of neuro-inflammation and neurodegeneration in the central nervous system (CNS), people with MS suffer from a wide range of sensory and motor symptoms that influence their quality of life [3]. People with MS (PwMS) can exhibit lower levels of muscle strength, speed, endurance, and cardiorespiratory fitness compared to healthy individuals [4]. Moreover, PwMS were reported to avoid physical activities believing that elevated body temperature worsens their symptoms [5]. However, recent studies have shown that physical exercises can positively affect the quality of life [6, 7], potentially by playing disease-modifying roles in PwMS, lessening depression [8, 9], fatigue [10, 11], paresthesia [12], and improving sexual dysfunction, emotion regulation, and subjective and objective sleep dimensions [13, 14].

The underlying biological mechanisms of MS are complex and not fully understood [15]. Dysregulation of the CD4^+^ T cells (T-helpers) remains the main immunological background of this disease [16]. T-helper 1 and T-helper 17 cells are aberrantly found in the CNS lesions, cerebrospinal fluid (CSF), and blood of people with MS [17]. These cells are associated with elevated inflammatory cytokines, such as interleukin-6 (IL-6), interferon-gamma (INF-γ), tumor necrosis factor-alpha (TNF-α), IL-17, and IL-22 in MS, both leading to the blood–brain barrier (BBB) breakdown and astrocyte and microglia activation [18–20]. In contrast, T-helper 2 cells, suppressors of microglial activation, are declined in MS; and reduced T-helper 1/T-helper 2 ratios in the CSF of PwMS enhance the neuro-inflammation in this disease [20, 21].

In multiple sclerosis, the rise in pro-inflammatory cytokines in blood and CSF accelerates the demyelination and axonal damage in the CNS [22, 23]. Among the main pro-inflammatory cytokines, including IL-1β, IL-6, and TNF-α [24], the role of IL-1 β is reported to be limited compared to the other two. A study showed that the role of IL-1 signaling in immune cells is redundant for the pathogenesis of experimental autoimmune encephalomyelitis (EAE), a murine model of MS [25]. On the contrary, the blockade of IL-6 and TNF-α in EAE suppresses disease development [26, 27]. Furthermore, high levels of IL-6 in the cerebrospinal fluid correlate with reduced synaptic plasticity with clinical expression of brain damage [28]. Physical exercise is reported to suppress CNS IL-6 production and thus inhibit microglial activation [28, 29]. On the other hand, muscle-derived IL-6 during physical activities stimulates anti-inflammatory cytokines (e.g., IL-10) and, therefore, inhibits the effects of tumor necrosis factor-alpha, the other pro-inflammatory cytokine [30]. TNF-α levels are also elevated in MS patients and are associated with MS severity [31].

The origin of these cytokines is variable in the body, and distinct sources determine different functions in cytokines; for example, it is suggested that the elevated IL-6 levels are mainly due to its production in skeletal muscle, brain, and peri‑tendinous tissues [32]. Contrary to immunologic cell-derived IL-6, muscle-derived IL-6 exhibits anti-inflammatory features, as it suppresses T-helper 1 cells and its interferon-gamma production and induces the production of IL-10 and Il-4 [33]. Furthermore, the elevation of muscle TNF-α is reported to have regenerative effects on muscles through activating satellite cells [34].

Exercise appears to influence the central nervous system (CNS) in a variety of ways. These include boosting cerebral blood flow, modulating endocannabinoids and neurotransmitters, influencing neuroendocrine responses, and CNS structural changes. [35–37] For instance, Prakash et al. [38] showed that aerobic exercise in PwMS decreased gray matter volume while maintaining the integrity of white matter. Several brain regions, including the hippocampus, thalamus, caudate, putamen, and pallidum, have been positively linked to the amount of moderate/vigorous physical exercise [39]. PwMS who completed a six-month, two-day-a-week weight exercise program had no significant reduction in brain atrophy, according to Kjølhede et al. [40]. Reflecting these structural findings, physical therapy and exercise seem to improve cognitive functioning, including memory, learning, and information processing [41–45].

To date, there is no eligible biomarker to assess the effects of exercise on people with MS. Herein, in this systematic review and meta-analysis, we aimed to evaluate the acute and long-term impacts of physical activities on serum IL-6 and TNF-α, the well-known pro-inflammatory cytokines, in people with MS. We will also discuss whether the cytokine changes are related to disease progression and clinical outcomes in these people.

## Methods and materials

The Preferred Reporting Items for Systematic Reviews and Meta-Analyses (PRISMA 2020) guidelines [46] were followed for this meta-analysis.

### Information source and search strategy

We performed an online search via PubMed, EMBASE, SCOPUS, Web of Science, and Cochrane Library until September 2021, aiming to identify original studies investigating IL-6 and TNF-α changes after exercise in MS patients and controls. There were no language or date restrictions. Results from PubMed and Embase were retrieved using Medical Subject Headings (MeSH) and Emtree, respectively. Additionally, we searched the reference lists of relevant papers for other publications that met the criteria. Our search keywords are described in the Additional file 1.

### Selection criteria

Studies were included if (1) they were peer-reviewed clinical trial studies, (2) IL-6 or TNF-α blood levels were measured quantitatively using enzyme-linked immunoassays (ELISA) or other assays, (3) IL-6 or TNF-α measured before and after an exercise intervention, and (4) the absolute values of the IL-6 or TNF-α markers were either given within the manuscript or provided by the authors of the original study for performing the meta-analysis. Exclusion criteria were (1) pediatric MS and (2) case reports, case series, letters, commentaries, abstracts, protocols, review articles, and animal and in vitro studies. Two authors (P.S and H.S) independently completed the screening and eligibility assessment. In case of disagreement, the two authors discussed and settled the conflict.

### Data extraction

Two reviewers independently extracted (1) bibliographic information (study title, year of publication, first author, study type, and country), (2) demographic and clinical features of the sample (number of patients and controls, age, sex, disease duration, mean expanded disability status scale [EDSS] score), (3) methodological details (diagnostic criteria, characteristics of the ELISA or other assay), and (4) levels of the IL-6 or TNF-α before and after the intervention in either MS or control group. We contacted the studies' corresponding authors for more information if the absolute values of the levels of IL-6 or TNF-α were not included in the published article. The inter-rater reliability between reviewers was calculated using the kappa coefficient [47].

### Study quality assessment

The methodological quality of the included studies was rated by two reviewers (P.S and H.S) separately, based on the PEDro scale [48]. PEDro is a trustworthy and valid checklist consisting of 11 items as follows: (1) eligibility criteria, (2) random allocation, (3) concealed allocation, (4) baseline comparability, (5) masked participants, (6) masked therapists, (7) masked assessors, (8) adequate follow-up, (9) intention to treat analysis, (10) between-group comparison, and (11) point estimates and variability. As the eligibility criterion item does not count to the overall score, each study gets a score from 0 to 10. We categorized studies based on their PEDro score; below 4 as "poor" quality, a score between 4 and 5 indicating "fair" quality, a score of 6 to 8 regarded to be of "good" quality, and a score of 9 to 10 indicating "excellent" quality. Any differences were addressed by discussion between the reviewers.

### Statistical analysis

We calculated a standardized mean difference (SMD) (Hedges' g), and 95% confidence interval (CI) for each between-group comparison as the included studies were done in a 17-year span and probably used assays with different sensitivity. The SMD values ≤ 0.2, 0.2–0.8, and ≥ 0.8 denoted small, moderate, and large effect sizes, respectively. Meta-analyses were done for comparisons for which findings from at least three separate datasets were available.

If the values reported in the manuscript were given as a median and interquartile range (IQR) or median and range, and we were not able to retrieve the mean ± standard deviation (SD) from the authors, we used statistical methods suggested by Luo et al. [49] and Wan et al. [50] to convert these values.

To assess heterogeneity between studies in the between-group meta-analyses, we used Cochrane's Q-test and the *I*^2^-index. The *I*^2^-indices of ≤ 25%, 26–75%, and 75% ≤ represented low, moderate, and high heterogeneity degrees, respectively. In terms of the heterogeneity tests, the *p-value* < 0.1 was considered significant. We utilized random effect models according to the DerSimonian and Laird method. Random-effects models are preferred if significant heterogeneity is expected, as they account for variable underlying effects in estimates of uncertainty, including both within- and between-study variance. We visualized the results of the meta-analysis as forest plots.

To further assess the causes of heterogeneity, we conducted a sensitivity analysis to identify influential cases for meta-analyses with significant heterogeneity, including ten or more studies. Each time we omitted one study and recalculated the effect size (leave-one-out Analyses). To reduce the heterogeneity among individual studies, we conducted a subgroup analysis based on the type of intervention used in each study.

Publication bias was initially assessed by visual observation of the degree of funnel plot asymmetry. Then, we used Egger's bias test to objectively confirm the visual perception from the funnel plot. A *p-value* < 0.1 was considered as evidence of publication bias. Funnel plots and Egger's plots are available. When there was evidence of publication bias, we adjusted the effect sizes using the trim-and-fill method.

All computations and visualizations were carried out using R version 4.0.4 (R Core Team [2020]. R: A language and environment for statistical computing. R Foundation for Statistical Computing, Vienna, Austria) and STATA 16 (StataCorp. 2019. Stata Statistical Software: Release 16. College Station, TX: StataCorp LLC) for metaregression and Egger's plots. We used following packages: “meta” (version 4.17–0), “metafor” (version 2.4–0), “dmetar” (version 0.0–9), and “tidyverse” (version 1.3.0). All forest plots and the drapery plot were designed using R. A *p-value* of < 0.05 was considered statistically significant.

## Results

### Selection of studies

**Fig. 1:**
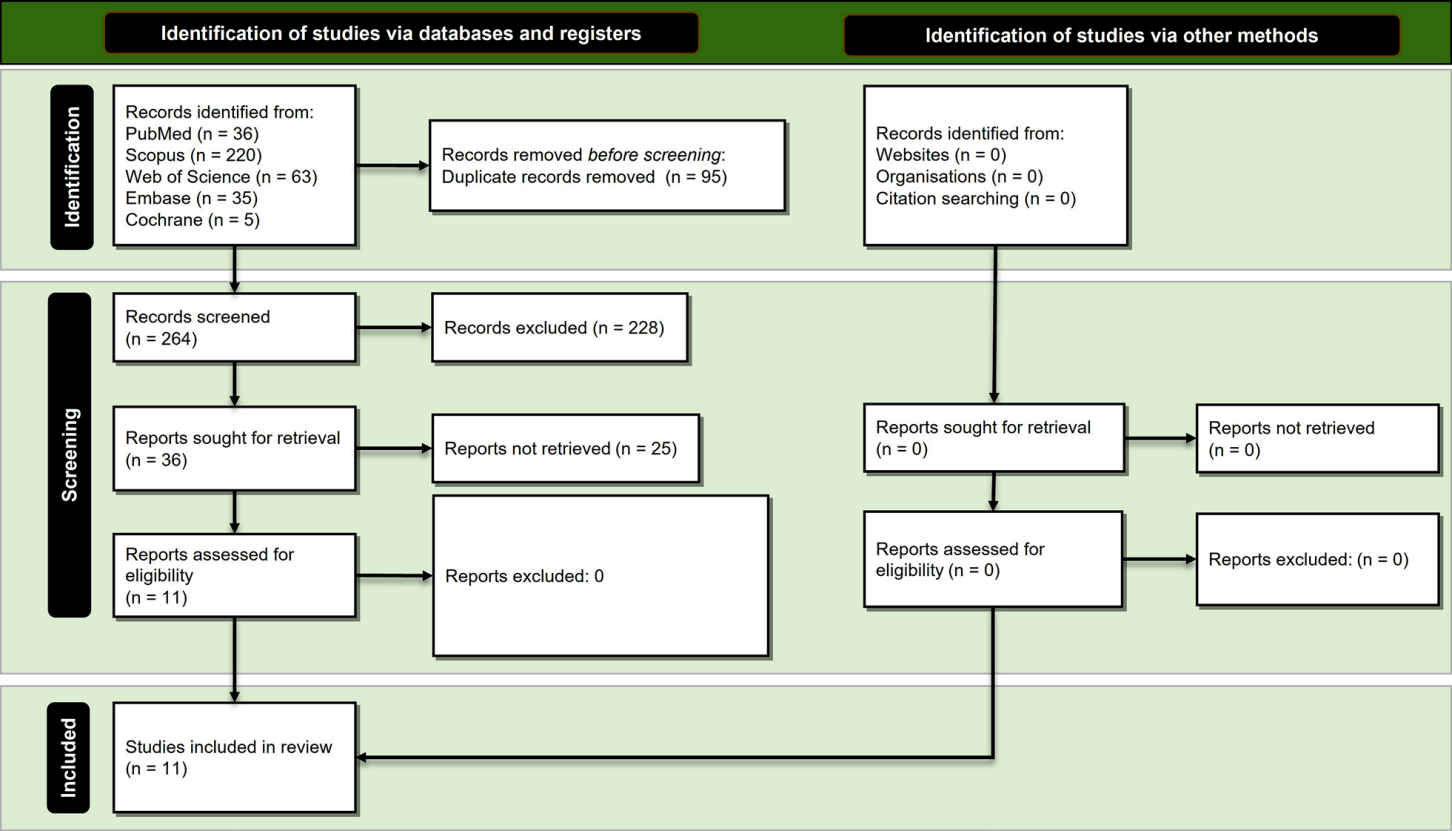
Study selection process according to the Preferred Reporting Items for Systematic Reviews and Meta- Analyses (PRISMA) guideline

The search strategy retrieved a total yield of 359 studies. After the removal of duplicates, 264 studies remained. In the next step, titles and abstracts were screened regarding their relevancy to the systematic review topic. After the exclusion of 228 irrelevant studies, we identified 36 potentially eligible studies. We checked the full text of these studies, and finally, 11 original clinical trials met the criteria to be included in the meta-analysis [51–61]. No further studies that were appropriate for inclusion were identified via hand searching and checking references. Figure 1 illustrates the process of study selection according to the PRISMA guideline. The agreement between the two independent reviewers for study selection was great for both titles/abstracts (kappa = 1.00; percentage agreement = 99.92%) and full text (kappa = 1.00; percentage agreement = 100%).

### Study characteristics

Table 1 shows the characteristics of the studies included in the meta-analysis. Levels of IL-6 were measured in nine studies [51–59], of which five studies had control groups [51, 54, 55, 58, 59]. Regarding TNF-α levels, six studies assessed its levels [52, 53, 55, 56, 60, 61], of which three investigated controls as well [55, 60, 61].

**Table 1:** Characteristics of the included studies

Studies						Patients					Controls			
Study	Country	Exercise Protocol	Type of Exercise	Source	Assessed Cytokines	N	Mean Age (years)	Male, %	Mean Disease Duration	Mean EDSS score	Type of Controls	N	Mean age (years)	Male, %
Berkowitz et al., 2019	Israel	A session of training for 2 h	Aerobic exercises	Serum	IL-4, IL-6, IL-10, IL-17A, IFN-ɣ, TNF-α	14	33.80	0	-	-	Healthy people who exercised	9	28.3	0
Devasahayam et al., 2020	Canada	Training for ten weeks (3x/week)	Aerobic exercises	Serum	IL-6, BDNF	7	-	-	-	-	-	-	-	-
Devasahayam et al., 2021	Canada	A session of graded exercise test (GXT)	Aerobic exercises	Serum	IL-6, BDNF	14	54.07	28%	16.57	-	Healthy people who exercised	8	50.71	37%
Donia et al., 2019	Canada	A session of training for 1 h	Aerobic exercises	Serum	IL-6, TNF-α, IFN-ɣ, IL-1RA	13	57.20	23%	-	-	-	-	-	-
Faramarzi et al., 2020	Iran	Training for 12 weeks (3x/week)	Combined aerobic and resistance exercises	Plasma	IL-6, IFN-ɣ	46	-	0%	-	-	MS patients who didn't exercise	43	-	0%
Kierkegaard et al., 2016	Sweden	Training for 12 weeks (2x/week)	Resistance exercises	Serum	IL-1RA, IL-4, IL-5, IL-6, IL-7, IL-8, IL-12p70, IL-13, IL-17	17	-	-	-	-	-	-	-	-
Kordi et al., 2014	Iran	Training for 8 weeks (4x/week)	Combined aerobic and resistance exercises	Serum	IL-10, TNF-α	27	33.68	-	-	1.78	MS patients who didn't exercise	8	33.63	-
Mokhtarzade et al., 2021	Iran	Training for 6 months (5x/week)	Combined aerobic and resistance exercises	Serum	IL-10, TNF-α	21	35.06	28%	4.35	2.14	MS patients who didn't exercise	21	36.38	23%
Raisi et al., 2018	Iran	Training for 12 weeks (3x/week)	Combined aerobic and resistance exercises	Serum	IL-6	48	-	0%	-	-	MS patients who did only stretching trainings	48	-	0%
Schulz et al., 2004	Germany	A session of training for 30 min	Aerobic exercises	Plasma	IL-6, sIL-6R, BDNF, NGF	15	39	26%	11.40	2.30	MS patients who didn't exercise	13	40	38%
White et al., 2006	United States	Training for 8 weeks (2x/week)	Resistance exercises	Serum	IL-2, IL-4, IL-6, IL-10, IFN-ɣ, TNF-α	10	47	0%	-	3.80	-	-	-	-

### Quality assessment

The median total PEDro score was 5 (IQR = 1; mean ± SD = 5.5 ± 0.8; range: 4 to 7) out of 10, indicating that the included studies were of good quality overall (Table 2). All studies passed the following criteria: (1) Between-group statistical comparison and (2) point estimates and variability. Moreover, neither the participants nor the raters were blinded in any of the included investigations.

**Table 2:** Quality assessment of the included studies, based on the PEDro scale

	Berkowitz et al., 2019	Devasahayam et al., 2020	Devasahayam et al., 2021	Donia et al., 2019	Faramarzi et al., 2020	Kierkegaard et al., 2016	Kordi et al., 2014	Mokhtarzade et al., 2021	Raisi et al., 2018	Schulz et al., 2004	White et al., 2006
Eligibility criteria	*	*	*	*	*	*		*	*		
Random allocation					*		*	*	*	*	
Concealed allocation					*		*	*	*	*	
Baseline comparability	*	*	*	*		*	*	*		*	*
Masked participants											
Masked therapists											
Masked assessors											
Adequate follow-up	*		*	*	*	*			*	*	*
Intention to treat analysis				*				*			*
Between-group statistical comparison	*	*	*	*	*	*	*	*	*	*	*
Point estimates and variability	*	*	*	*	*	*	*	*	*	*	*
Total score	5	4	5	6	6	5	5	7	6	6	5

### Meta-analysis

All statistical indices for within- and between-group meta-analysis are reported in Table 3. Overall, for pre–post-comparisons within individuals with MS, IL-6 did neither change after acute nor regular exercise. For TNF-α, overall concentrations decreased from pre- to post-intervention, though no change in TNF-α concentrations was observed for acute exercise when considered separately. Interestingly, regular exercise resulted in significant differences from pre- to post-intervention. Compared to controls, in individuals with MS, neither IL-6 nor TNF-α concentrations decreased or increased after acute and regular exercise.

**Table 3:** Results of within- and between-group meta-analyses.

Comparison	Subgroup	No. studies	No. Cases	No. Controls	Meta-analysis					Heterogeneity		
					Effect size	95% Confidence interval (%)	*p*		Eggers	I^2^ (%)	*Q*	*p*
Pre–Post												
IL-6	Overall	9	184	NA	−0.0925	−0.2976; 0.1125	0.3765		0.45	0.0%	3.69	0.8843
	Acute Exercise	4	56		−0.0724	−0.4459; 0.3010	0.7037	0.8997		0.0%	2.98	NA
	Regular Exercise	5	128		−0.1012	−0.3465; 0.1442	0.4189			0.0%	0.69	
TNF-α	Overall	6	102	NA	−0.5162	−0.9195; -0.1129	***0.0121***		0.27	48.6%	9.73	***0.0833***
	Acute Exercise	2	27		−0.2190	−0.7551; 0.3171	0.4233	0.2591		0.0%	0.28	NA
	Regular Exercise	4	75		−0.6705	−1.2429; -0.0982	***0.0217***			63.7%	8.26	
MS-Control												
IL-6	Overall	5	137	121	−0.0888	−0.3351; 0.1575	0.4798		0.18	0.0%	2.66	0.6162
	Acute Exercise	3	43	30	0.1023	−0.3673; 0.5719	0.6694	0.3579		0.0%	0.61	NA
	Regular Exercise	2	94	91	−0.1623	−0.4750; 0.1503	0.3088			14.4%	1.17	
TNF-α	Overall	3	62	38	−0.2364	−0.6551; 0.1824	0.2686		0.23	0.0%	0.60	0.7410
	Acute Exercise	1	14	9	0.0108	−0.8266; 0.8482	NA	0.5041		NA	NA	NA
	Regular Exercise	2	48	29	−0.3188	−0.8023; 0.1648	0.1963			0.0%	0.15	

Within-group analysis: individuals with multiple sclerosis: pre- vs. post-comparisons for acute and regular exercise. Between-group analysis: individuals with vs. without multiple sclerosis; comparisons for acute and regular exercising

MS = multiple sclerosis; IL-6 = interleukin-6; TNF-α = Tumor Necrosis Factor-alpha. NA = Not Applicable

Significant *p-values* are in Bold

#### IL-6

##### Comparison of pre- and post-intervention IL-6 concentration

In nine studies, levels of IL-6 were assessed (*N* = 184) before and after the exercise program. The combined mean ± SD of the age of participants was 46.59 ± 12.91 years, as reported by six studies (*n* = 73) [51, 52, 55–57, 59]. The cumulative number of female and male participants was 172 and 12, respectively, reported by eight studies [51, 52, 54–59]. Three studies reported the EDSS score of patients [51, 52, 57] (*n* = 32), and the combined EDSS mean ± SD was 3.72 ± 1.79. The combined mean ± SD of the disease duration was 14.84 ± 7.92 years (*n* = 39), reported by three studies [51, 57, 59].

**Fig. 2:**
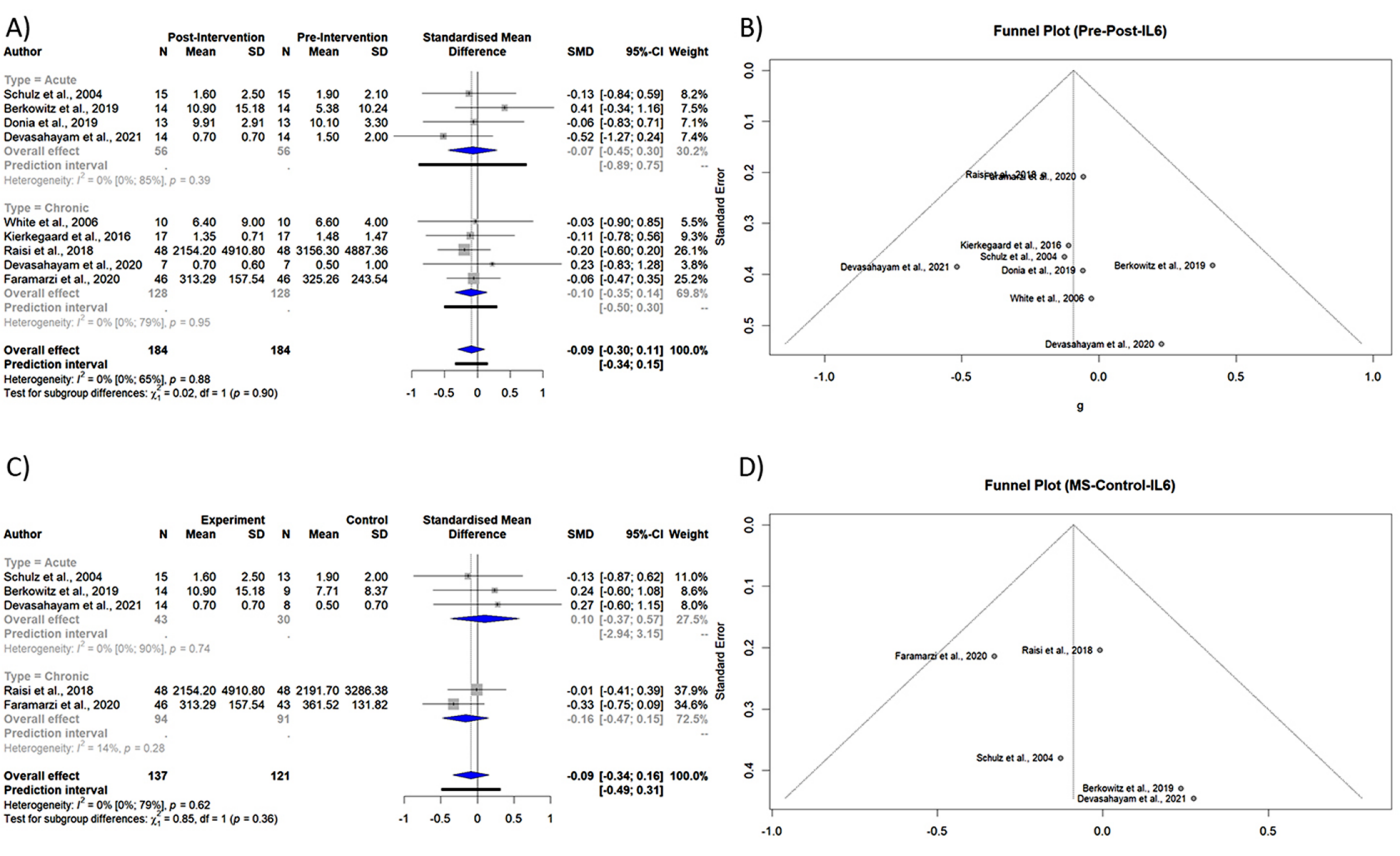
**A** Forest plot of subgroup meta-analysis of pre- and post-intervention levels of IL-6 **B** Funnel plot of meta-analysis of pre- and post-intervention levels of IL-6 **C** Forest plot of subgroup meta-analysis of PwMS vs. Control levels of IL-6 **D** Funnel plot of meta-analysis of PwMS vs. Control levels of IL-6

Meta-analysis showed that IL-6 levels were lower after exercise but did not reach statistical significance (Table 3, Fig. 2A). A drapery plot is used to represent the meta-analysis findings based on the study's *p-value* functions (*p-value* on the y-axis and the effect size on the x-axis), which are depicted in the Additional file 1 for all four meta-analyses.

The Egger's test (*p-value* = 0.45) and funnel plot (Fig. 2B) exhibited no evidence of publication bias. The Eggers' test does not indicate the presence of substantial funnel plot asymmetry. The heterogeneity between studies was not statistically significant (*p-value* = 0.8843).

##### Comparison of post-intervention IL-6 levels between PwMS and controls

In five studies, levels of IL-6 were compared between PwMS (*N* = 137) and controls (*N* = 121). The combined mean ± SD of participants' age was 42.21 ± 11.92 years reported by three studies (*n* = 43) [51, 55, 59]. The cumulative number of female and male participants was 177 and 8, respectively [51, 54, 55, 58, 59]. Just one study reported the EDSS score of the patients [59]. The combined mean ± SD of disease duration was 13.89 ± 7.19 years [51, 59].

No statistically significant difference was observed comparing post-intervention levels of IL-6 between PwMS and controls (Table 3, Fig. 2C).

The Egger's test (*p-value* = 0.18) and funnel plot (Fig. 2D) disclosed no evidence of publication bias. The Eggers' test does not indicate the presence of substantial funnel plot asymmetry. The heterogeneity between studies was not statistically significant (*p-value* = 0.6162).

#### TNF-α

##### Comparison of pre- and post-intervention TNF-α concentration

In six studies, levels of TNF-α were determined before and after the exercise program in PwMS (*N* = 102). The combined mean ± SD of the age of participants was 32.2 ± 12.08 years, as reported by five studies (*n* = 85) [52, 55, 56, 60, 61]. The cumulative number of female and male participants was 74 and 9, respectively [52, 55, 56, 61]. Three studies reported the EDSS score of the patients (*n* = 58) [52, 60, 61], and the combined EDSS mean ± SD was 2.25 ± 1.16. One study reported the disease duration time of the participants [61].

**Fig. 3:**
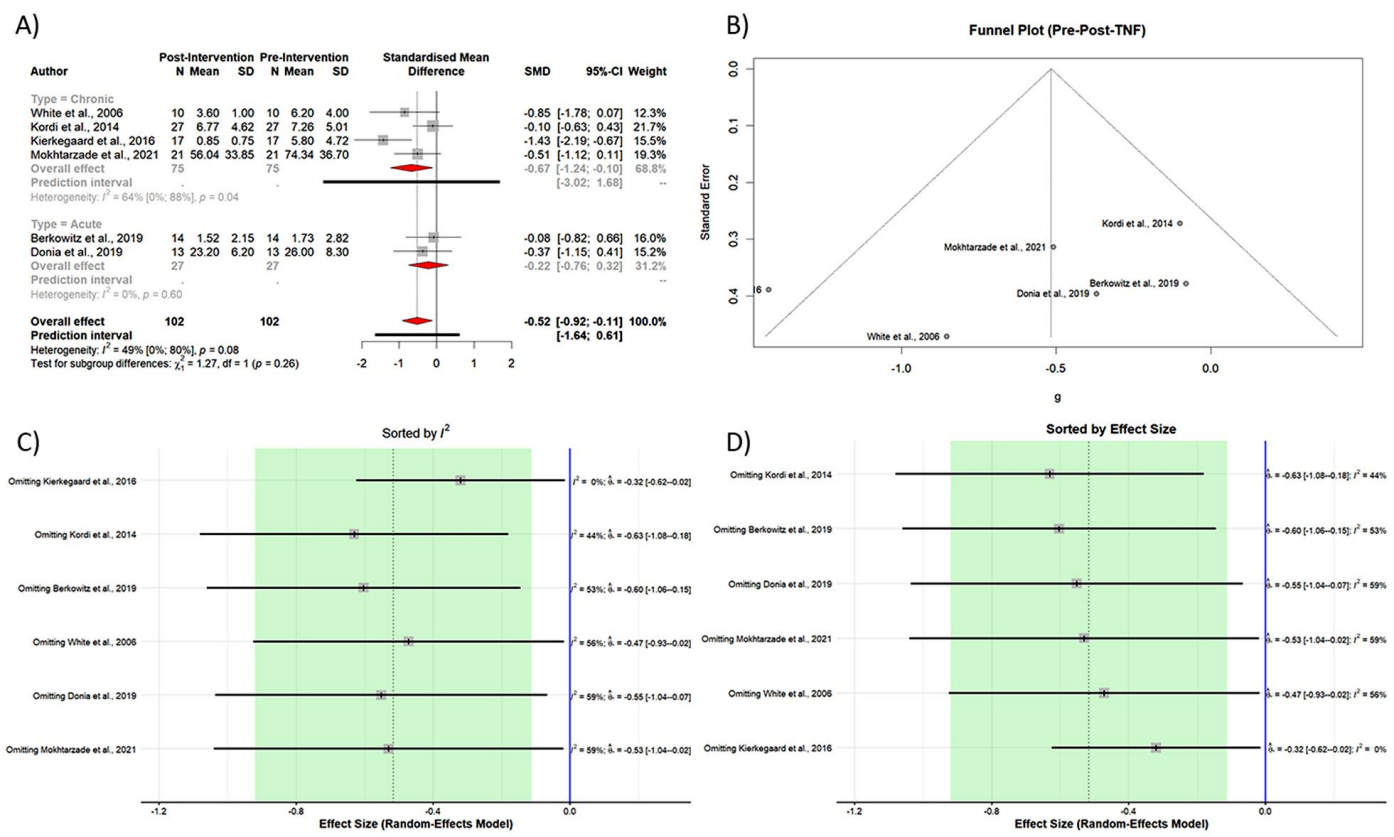
A Forest plot of subgroup meta-analysis of pre- and post-intervention levels of TNF-α B Funnel plot of meta-analysis of pre- and post-intervention levels of TNF-α C and D Result of sensitivity analysis

TNF-α levels were lower after exercise intervention, but not achieving statistical significance (SMD −0.5161, 95% CI [−1.0488; 0.0166], *p-value* = 0.0551, test of heterogeneity: *I*^2^ = 48.6%, *p-value* = 0.0836 (Table 3, Fig. 3A).

The Egger's test (*p-value* = 0.27) and funnel plot (Fig. 3B) showed no evidence of publication bias. The Eggers' test does not indicate the presence of substantial funnel plot asymmetry. The heterogeneity between studies was statistically significant (*p-value* = 0.0836).

Sensitivity analysis (leave-one-out analysis) showed that the effect size remained significant after omitting each study, and the heterogeneity did significantly reduce (Fig. 3C and D).

##### Comparison of post-intervention TNF-α levels between PwMS and controls

In three studies, the levels of TNF-α were compared between PwMS (*N* = 62) and controls (*N* = 48). The combined mean ± SD of the age of participants was 34.17 ± 7.9 years for 62 reported patients of three studies [55, 60, 61]. The cumulative number of female and male participants was 29 and 6, respectively, reported by two studies [55, 61]. Two studies reported the EDSS score of the patients [60, 61], and the combined EDSS mean ± SD was 1.93 ± 0.94 for 48 reported patients. One study reported the disease duration time of the participants [61].

**Fig. 4:**
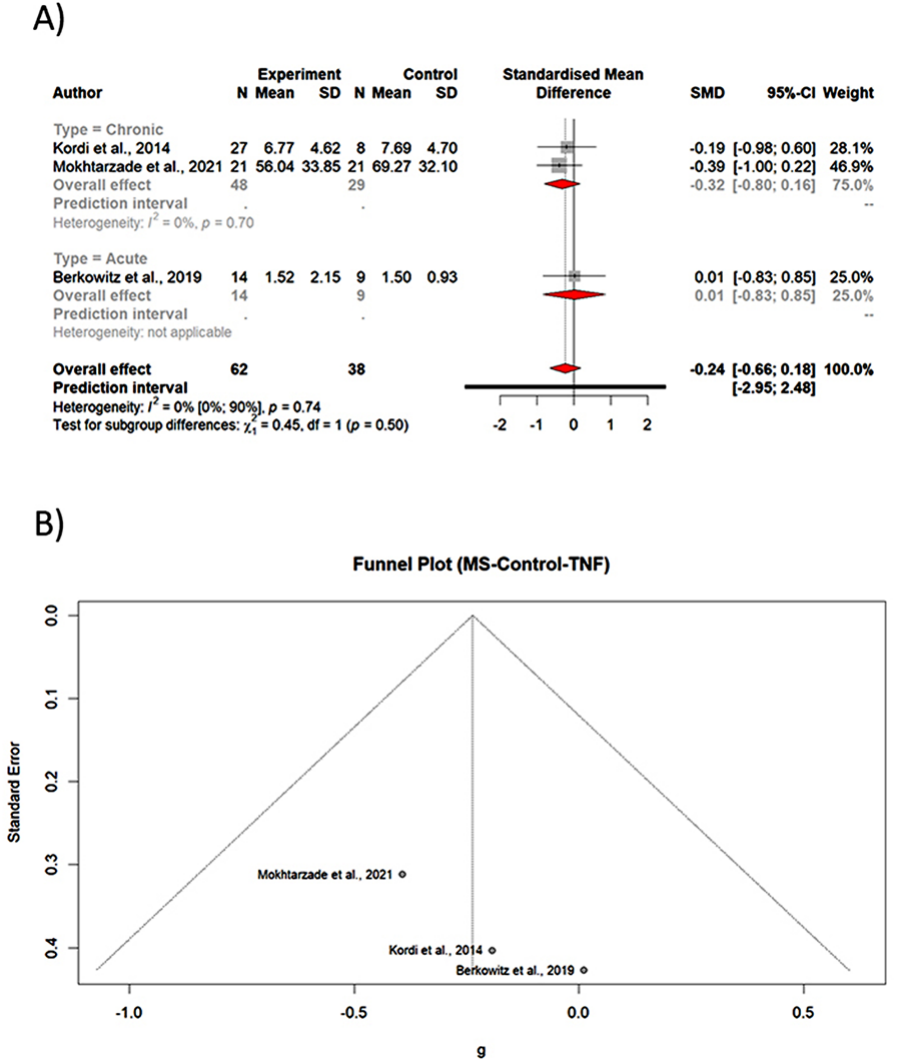
A Forest plot of subgroup meta-analysis of PwMS vs. Control levels of TNF-α B Funnel plot of meta-analysis of PwMS vs. Control levels of TNF-α

No statistically significant difference was observed comparing post-intervention levels of TNF-α between PwMS and controls (Table 3, Fig. 4A).

The Egger's test (*p-value* = 0.23) and funnel plot (Fig. 4B) demonstrated no evidence of publication bias. The Eggers' test does not indicate the presence of substantial funnel plot asymmetry. The heterogeneity between studies was not statistically significant (*p-value* = 0.7410).

## Discussion

To the best of our knowledge, the current review is the first meta-analysis evaluating the acute and long-term impacts of exercise on serum IL-6 and TNF-α in PwMS and compared to healthy controls. Results can be summarized in five points: First, in PwMS, acute and regular exercise had no impact on IL-6 levels. Second, for TNF-α, overall concentrations decreased from pre- to post-intervention; however, third, no change in TNF-α concentrations was observed for acute exercise when considered separately. Fourth, regular exercise resulted in significant differences from pre- to post-intervention. Lastly, compared to controls, in PwMS, IL-6 and TNF-α concentrations did not change after acute and regular exercise. Given these, the present results significantly add to the current literature: Both acute and regular exercise does either not impact or favorably impact IL-6 and TNF-α levels in PwMS; as such, both acute and regular exercises do not further deteriorate the immune system; this is disturbed in PwMS.

Blood and CSF cytokine alterations have been reported in MS patients, but the results are not consistent given the heterogeneity of studies. Most studies involved a small number of MS patients, not controlling for the phase of the disease (e.g., remission vs. relapse/acute, relapsing–remitting vs. progressive) [62–67]. According to a recent meta-analysis of these studies, TNF-α was significantly higher in PwMS compared to healthy controls (*p-value* < 0.001), but differences in IL-6 blood concentrations in PwMS and healthy controls were not (*p-*value = 0.064) [68]. Similarly, CSF levels of TNF-α was significantly higher in PwMS, unlike IL-6 [68].

Exercise has been associated with changes in the peripheral levels of cytokines. For example, Ostrowskie et al. demonstrated that IL-6 plasma concentration of athletes increases significantly after 2.5 h of treadmill running, while TNF-α remains unchanged [69]. Kouvelioti et al. observed significant elevations of IL-1β, IL-6, and TNF-α serum levels after high-intensity interval running and cycling [70]. Townsend et al. assessed the circulating levels of TNF-α after heavy resistance exercise in men. They reported that TNF-α elevates immediately after resistance exercise but decreases at 24 and 48 h after that [71]. Different exercise protocols, including type, duration, and intensity alongside distinct measurement methods, and targeted population might explain differences among these studies [70] [72, 73]. Age and gender are other factors contributing to different post-exercise cytokine changes [74].

Several studies have also investigated exercise-induced cytokine alterations in neurodegenerative diseases [75–77]. For example, plasma levels of IL-6, but not TNF-α, increased after a 16-week duration of moderate-to-high-intensity aerobic physical exercise in Alzheimer’s disease patients [78]. Conversely, TNF-α levels decreased [79] or remained unaltered in PD patients after 8-week course of aerobic exercise [80].

In contrast to these studies in healthy subjects and people with neurodegenerative disorders, PwMS did not change their TNF-α and IL-6 blood levels after physical activity. The impaired aerobic capacity in PwMS could explain the observed differences between healthy people and PwMS regarding cytokine changes. The decreased oxygen transportation/mitochondrial phosphorylation in MS is associated with disease severity and results in different cytokine alterations [81].

Given that systemic IL-6 regulation alterations may be significant in the establishment of central nervous system lesions [82], reductions in this cytokine may have substantial clinical outcomes in people with MS. Previous research indicates that excessively high IL-6 concentrations in the peripheral may cause excess inflammation, which may aggravate disease activity in MS [83]. Moreover, increased IL-6 may interfere with microbial pathogen clearance [83] and contribute in T-cell activation, thereby contributing to MS disease processes [83, 84]. Furthermore, plasma IL-6 levels may be a marker of skeletal muscle-controlled metabolic regulation. IL-6 has both paracrine and endocrine effects. IL-6 may influence the release of more IL-6 from local skeletal muscle [85], or it may circulate and influence hepatic glucose release [86]. Also, when glucose reliance reduces, resting basal IL-6 concentrations have been reported to decrease with training [87]. As a result, reductions in IL-6 may represent a training response and may reflect metabolic alterations. More investigations that clearly determine the impact of variations in IL-6 in MS patients would be beneficial.

Recent studies demonstrate that TNF-α may be neuroprotective by increasing the proliferation of oligodendrocytes and stimulating remyelination [82, 88, 89], despite the fact that it has been connected to MS-related inflammatory demyelination [90–92]. In fact, intravenous anti-TNF-α medication proved ineffective in MS patients and may have worsened their symptoms [88, 93]. As a result, resolving TNF-α's paradoxical involvement in disease activity is challenging. One reason might be the occurrence of two distinct signaling pathways mediated by two distinct TNF-α receptors (p55 and p75) [88, 89]. Exercise may cause activation of the "good" inflammatory TNF-α-p75 receptor pathway, which stimulates cell growth and proliferation [88]. All of these data suggested that TNF-α plays a critical part in the disease progression of MS and that blocking its effects may lower the severity of MS symptoms. TNF-α inhibitors are being employed as an effective treatment option in a variety of autoimmune and inflammatory conditions [94]. Remarkably, none of the previous trials and investigations supported the use of anti-TNF drugs in MS.

As previously explained, PwMS hesitated to do exercises for a long time because they feared disease exacerbation [95]. Our study revealed that IL-6 and TNF-alpha levels insignificantly decrease after the acute phases of acute physical exercises. As the relapse phase of RRMS patients is associated with higher IL-6 and TNF-alpha levels than healthy controls [96], this might imply the anti-inflammatory effect of physical activity and its protection from disease exacerbation in patients. However, neurologists should take caution when recommending PwMS to exercise, as aggressive training could result in excess heat and injuries. These factors are associated with disease exacerbation in these people [97, 98]. If prescribed cautiously, physical activity can lead to a better quality of life in PwMS [99].

The limitations of the current meta-analysis reflect the limitations of the available studies investigating the effect of exercise on PwMS. Most studies enrolled small number of patients and involved short-term interventions (less than 26 weeks), with different types of exercise. Moreover, most recruited patients had low levels of disability (EDSS scores of < 4) and relapsing–remitting MS, not presenting medical comorbidities, decreasing the potential generalizability of the findings.

Further investigations, including a more significant number of patients with diverse forms of MS, are necessary to confirm these findings. These approaches can improve the overall quality and scope of the evidence on MS rehabilitation research.

## Conclusion

Among adult individuals with MS, both acute and regular exercise did not deteriorate but positively impacted two cytokines, namely, IL-6 and TNF-α levels in PwMS. Given this, the present systematic review and meta-analysis support the benefit of acute and regular exercise among PwMS. According to the results of our study, on a molecular basis, exercise does not result in a disturbed and inflammatory immune system in PwMS, and even lead to a reduction in TNF-α. These findings are in contrary to the belief of some neurologists and patients.

## Supplementary Information

**Additional file 1. **Search Keywords.

## Data Availability

All of the data will be available for secondary analysis in necessary cases from the corresponding author through the email address.

## Abbreviations

PwMS: People with multiple sclerosis
MS: Multiple sclerosis
CNS: Central nervous system
TNF-α: Tumor necrosis factor-alpha
IL-6: Interleukin-6
ELISA: Enzyme-linked immunosorbent assay
EDSS: Expanded disability status scale
SMD: Standardized mean difference
IQR: Interquartile range
SD: Standard deviation
