# Supplementary material for: IL-6 and TNF-α responses to acute and regular exercise in adult individuals with multiple sclerosis (MS): a systematic review and meta-analysis
Source: Eur J Med Res. 2022 Sep 26;27:185. doi: 10.1186/s40001-022-00814-9 (PMC9511785; doi:10.1186/s40001-022-00814-9)
Supplement: Supplementary file 1 — Additional file 1. Search Keywords. [file 40001_2022_814_MOESM1_ESM.docx]

**Supplementary material**

**Title: IL-6 and TNF-α Responses to Acute Exercise in People with Multiple Sclerosis (PwMS): a Systematic Review and Meta-analysis**

**Authors:** Parnian Shobeiri, Homa Seyedmirzaei, Antônio L. Teixeira, Serge Brand, Dena Sadeghi-Bahmani, Nima Rezaei*

***Corresponding author:** Nima Rezaei, MD, Ph.D., Research Center for Immunodeficiencies, Children’s Medical Center, Dr. Gharib St, Keshavarz Blvd, Tehran, Iran, E-mail: [rezaei_nima@yahoo.com](mailto:rezaei_nima@yahoo.com)

***Table of Contents***

Keywords2

1. **Keywords**

| #1 | (Sclerosis, Multiple[Title/Abstract]) OR (Sclerosis, Disseminated[Title/Abstract])) OR (Disseminated Sclerosis[Title/Abstract])) OR (MS[Title/Abstract])) OR (Multiple Sclerosis, Acute Fulminating[Title/Abstract])) OR (Multiple Sclerosis[Title/Abstract])) OR (Multiple Sclerosis[MeSH Terms])) OR (Multiple Sclerosis, Relapsing-Remitting[MeSH Terms])) OR (Multiple Sclerosis, Relapsing-Remitting[Title/Abstract])) OR (Multiple Sclerosis, Relapsing Remitting[Title/Abstract])) OR (Remitting-Relapsing Multiple Sclerosis[Title/Abstract])) OR (Multiple Sclerosis, Remitting-Relapsing[Title/Abstract])) OR (Remitting Relapsing Multiple Sclerosis[Title/Abstract])) OR (Relapsing-Remitting Multiple Sclerosis[Title/Abstract])) OR (Relapsing Remitting Multiple Sclerosis[Title/Abstract])) OR (Multiple Sclerosis, Acute Relapsing[Title/Abstract])) OR (Acute Relapsing Multiple Sclerosis[Title/Abstract])) OR (Multiple Sclerosis, Chronic Progressive[MeSH Terms])) OR (Multiple Sclerosis, Chronic Progressive[Title/Abstract])) OR (Chronic Progressive Multiple Sclerosis[Title/Abstract])) OR (Multiple Sclerosis, Progressive Relapsing[Title/Abstract])) OR (Multiple Sclerosis, Remittent Progressive[Title/Abstract])) OR (Remittent Progressive Multiple Sclerosis[Title/Abstract])) OR (Progressive Relapsing Multiple Sclerosis[Title/Abstract])) OR (Multiple Sclerosis, Secondary Progressive[Title/Abstract])) OR (Secondary Progressive Multiple Sclerosis[Title/Abstract])) OR (Multiple Sclerosis, Primary Progressive[Title/Abstract])) OR (Primary Progressive Multiple Sclerosis[Title/Abstract])) |
| --- | --- |
| #2 | (((((((((((((((((((((((((((((((Interleukin-6[MeSH Terms]) OR (Interleukin-6[Title/Abstract])) OR (Interleukin 6[Title/Abstract])) OR (IL6[Title/Abstract])) OR (B-Cell Stimulatory Factor 2[Title/Abstract])) OR (B-Cell Stimulatory Factor-2[Title/Abstract])) OR (Differentiation Factor-2, B-Cell[Title/Abstract])) OR (Differentiation Factor 2, B Cell[Title/Abstract])) OR (B-Cell Differentiation Factor-2[Title/Abstract])) OR (B Cell Differentiation Factor 2[Title/Abstract])) OR (BSF-2[Title/Abstract])) OR (Hybridoma Growth Factor[Title/Abstract])) OR (Growth Factor, Hybridoma[Title/Abstract])) OR (IFN-beta 2[Title/Abstract])) OR (Plasmacytoma Growth Factor[Title/Abstract])) OR (Growth Factor, Plasmacytoma[Title/Abstract])) OR (Hepatocyte-Stimulating Factor[Title/Abstract])) OR (Hepatocyte Stimulating Factor[Title/Abstract])) OR (MGI-2[Title/Abstract])) OR (Myeloid Differentiation-Inducing Protein[Title/Abstract])) OR (Differentiation-Inducing Protein, Myeloid[Title/Abstract])) OR (Myeloid Differentiation Inducing Protein[Title/Abstract])) OR (B-Cell Differentiation Factor[Title/Abstract])) OR (B Cell Differentiation Factor[Title/Abstract])) OR (Differentiation Factor, B-Cell[Title/Abstract])) OR (Differentiation Factor, B Cell[Title/Abstract])) OR (IL-6[Title/Abstract])) OR (Interferon beta-2[Title/Abstract])) OR (Interferon beta 2[Title/Abstract])) OR (beta-2, Interferon[Title/Abstract])) OR (B Cell Stimulatory Factor-2[Title/Abstract])) OR (B Cell Stimulatory Factor 2[Title/Abstract]) |
| #3 | ((((((((((Tumor Necrosis Factor-alpha[MeSH Terms]) OR (Tumor Necrosis Factor-alpha[Title/Abstract])) OR (Tumor Necrosis Factor alpha[Title/Abstract])) OR (Cachectin[Title/Abstract])) OR (Cachectin-Tumor Necrosis Factor[Title/Abstract])) OR (Cachectin Tumor Necrosis Factor[Title/Abstract])) OR (Tumor Necrosis Factor Ligand Superfamily Member 2[Title/Abstract])) OR (Tumor Necrosis Factor[Title/Abstract])) OR (TNF Superfamily, Member 2[Title/Abstract])) OR (TNFalpha[Title/Abstract])) OR (TNF-alpha[Title/Abstract]) |
| #4 | (Exercise[MeSH Terms]) OR (Exercise[Title/Abstract])) OR (Exercises[Title/Abstract])) OR (Physical Activity[Title/Abstract])) OR (Activities, Physical[Title/Abstract])) OR (Activity, Physical[Title/Abstract])) OR (Physical Activities[Title/Abstract])) OR (Exercise, Physical[Title/Abstract])) OR (Exercises, Physical[Title/Abstract])) OR (Physical Exercise[Title/Abstract])) OR (Physical Exercises[Title/Abstract])) OR (Acute Exercise[Title/Abstract])) OR (Acute Exercises[Title/Abstract])) OR (Exercise, Acute[Title/Abstract])) OR (Exercises, Acute[Title/Abstract])) OR (Exercise, Isometric[Title/Abstract])) OR (Exercises, Isometric[Title/Abstract])) OR (Isometric Exercises[Title/Abstract])) OR (Isometric Exercise[Title/Abstract])) OR (Exercise, Aerobic[Title/Abstract])) OR (Aerobic Exercise[Title/Abstract])) OR (Aerobic Exercises[Title/Abstract])) OR (Exercises, Aerobic[Title/Abstract])) OR (Exercise Training[Title/Abstract])) OR (Exercise Trainings[Title/Abstract])) OR (Training, Exercise[Title/Abstract])) OR (Trainings, Exercise[Title/Abstract])) |
